# Supplementary material for: The perceived impact of homelessness on health during pregnancy and the postpartum period: A qualitative study carried out in the metropolitan area of Nantes, France
Source: PLoS One. 2023 Feb 1;18(2):e0280273. doi: 10.1371/journal.pone.0280273 (PMC9891509; doi:10.1371/journal.pone.0280273)
Supplement: S1 Fig — (PDF) [file pone.0280273.s002.pdf]

Coding tree.

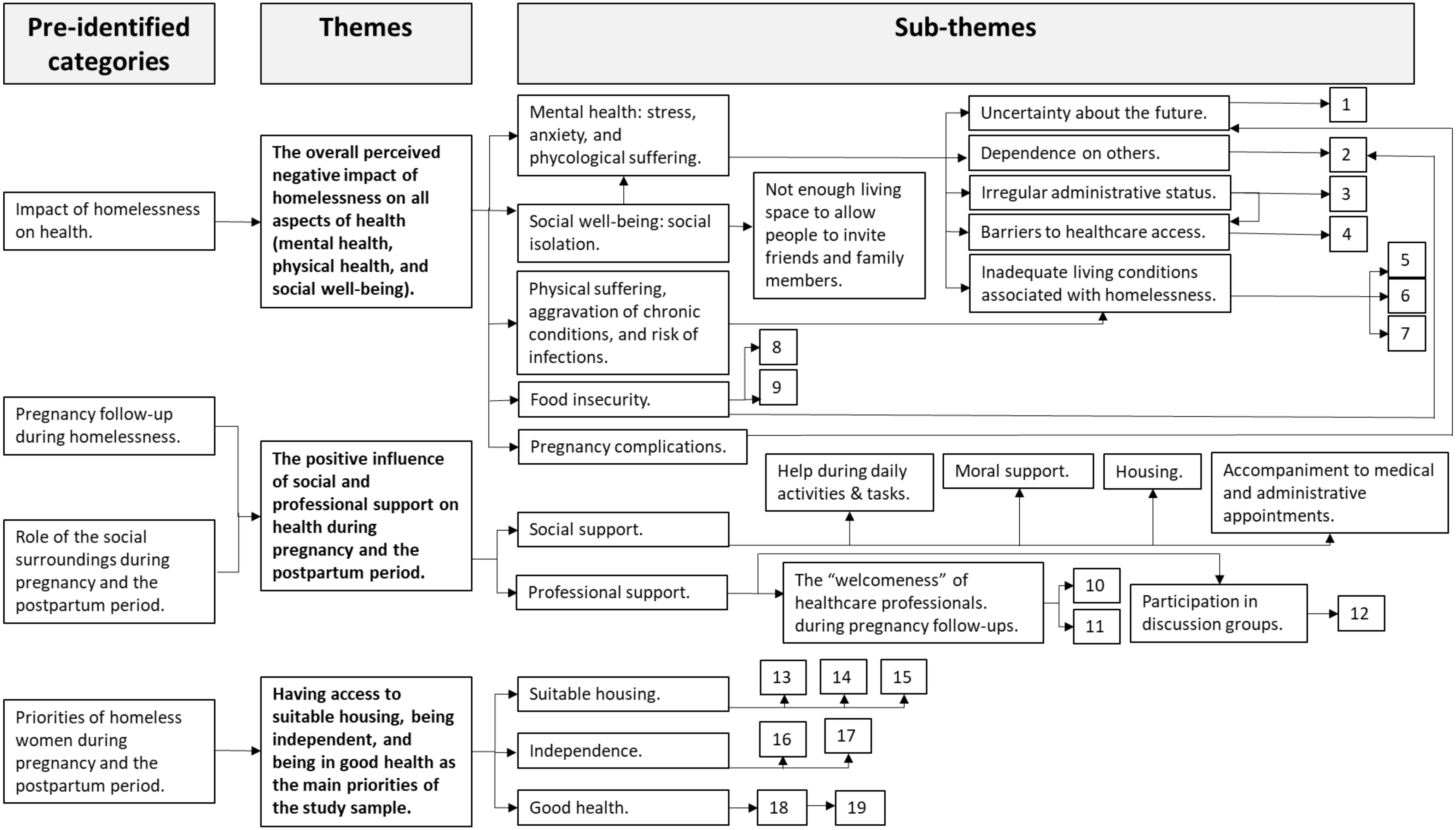

Pre-identified categories
Themes
Sub-themes
Impact of homelessness on health.
Pregnancy follow-up during homelessness.
Role of the social surroundings during pregnancy and the postpartum period.
Priorities of homeless women during pregnancy and the postpartum period.
The overall perceived negative impact of homelessness on all aspects of health (mental health, physical health, and social well-being).
The positive influence of social and professional support on health during pregnancy and the postpartum period.
Having access to suitable housing, being independent, and being in good health as the main priorities of the study sample.
Mental health: stress, anxiety, and phycological suffering.
Social well-being: social isolation.
Physical suffering, aggravation of chronic conditions, and risk of infections.
Food insecurity.
Pregnancy complications.
Not enough living space to allow people to invite friends and family members.
Uncertainty about the future.
Dependence on others.
Irregular administrative status.
Barriers to healthcare access.
Inadequate living conditions associated with homelessness.
1
2
3
4
5
6
7
8
9
Social support.
Professional support.
Help during daily activities & tasks.
Moral support.
Housing.
Accompaniment to medical and administrative appointments.
The “welcomeness” of healthcare professionals. during pregnancy follow-ups.
10
11
Participation in discussion groups.
12
Suitable housing.
Independence.
Good health.
13
14
15
16
17
18
19

Legend:

1. Housing instability.
2. Problems with local authorities.
3. Financial precarity.
4. Barriers in obtaining a health insurance.
5. Discomfort.
6. Low hygiene.
7. Difficult access to water, electricity, and heating.
8. Lack of cooking and storing facilities.
9. Food distributions of poor quality and not adapted to health and cultural needs.
10. Being informed by healthcare professionals.
11. Having an answer to questions related to pregnancy, childbirth, and the newborn’s well-being.
12. Sharing information, expertise, and doubts with the group.
13. Having access to basic facilities, such as water, electricity, and toilets.
14. Having enough space.
15. Having access to a kitchen.
16. Having access to a job.
17. Having a regular administrative status.
18. Having a healthcare coverage.
19. Being able to access the healthcare system.
