# Supplementary material for: The perceived impact of homelessness on health during pregnancy and the postpartum period: A qualitative study carried out in the metropolitan area of Nantes, France
Source: PLoS One. 2023 Feb 1;18(2):e0280273. doi: 10.1371/journal.pone.0280273 (PMC9891509; doi:10.1371/journal.pone.0280273)
Supplement: Attachment — Submitted filename: Response to reviewers.docx [file pone.0280273.s003.docx]

To editor:

Dear editor, the authors are deeply thankful for your consideration and for the interest you have shown for our work. We have addressed below the points raised during the review process and we have corrected the manuscript as suggested.

Best regards,

Dr Giulio Borghi

Journal requirements:

1. We have ensured that our manuscript meets PLOS ONE’s publication criteria.
2. We have provided additional details about how oral consent was obtained in lines 108-111.
3. The minimal data needed to support our study results have been integrated in the “Results” section of the manuscript. Nevertheless, as detailed in the data availability statement, the full interview transcripts are not available for the following ethical reasons:
   - The interview transcripts contain sensible information that, when crossed, could easily lead to the identification of participants (dates, names, addresses, specific life events, country of birth, languages spoken).
   - The relatively small group of homeless women being pregnant or in the postpartum period in the metropolitan area of Nantes, could easily lead to the identification of participants.
   - The de-identification or anonymization of the transcripts is not possible due to the qualitative approach adopted in this research. As a matter of fact, the unique life stories shared by participants could easily lead to their identification if considering the geographical area included in the study.

We assure you that none of the unacceptable data access restrictions cited in PLOS ONE’s guidelines apply to our case.

Additional editor comments:

\# Abstract

1. The study objective was changed as follows: “The objective of this study was to describe the overall perceived impact of homelessness on health during pregnancy and the postpartum period, prioritizing women’s perspective through a qualitative research approach.” (lines 24-16).

\# Introduction

1. We changed the citations’ format following the Journal’s guidelines.
2. More recent data has been inserted in the manuscript as requested. The new sentence is the following “In the specific case of France, the last estimations made by the French Court of Auditors, had shown an increase in homelessness of nearly 110% between 2012 (143,000 people) and 2019 (300,000 people).” (lines 48-50).

\# Method

1. Materials was removed as requested.
2. All the information of the qualitative approach used in our study can be found in the pages listed in the “study design” and “analysis and findings” sections of the COREQ checklist that we have added in the supporting information (S1 Table). Data was collected through one-time individual semi-structured interviews, as detailed in line 113.
3. The explanation on how study participants were selected in respect to age and mental health problems has been detailed in lines 83-88.
4. No incentives for study participants for time compensation were offered.
5. The positive evaluation of the discussion groups organized by the CHU of Nantes was freely evoked by participants during the interview. Our study did not aim to evaluate the satisfaction of those discussion groups. Nevertheless, interviewees were able to share their pregnancy follow-up experience without any restriction, either if it was positive or negative, and to share the experiences that mattered the most to them.

#Discussion

1. Gross modification and restructuring of the discussion have been done, focusing on the study population, as suggested.

To reviewers:

Dear reviewers, all the authors deeply appreciated the interest you have in the topic. We are thankful for the interesting insights you have highlighted in your reviews, as they allowed us to improve the quality and clarity of our manuscript. We have addressed below each of the points you have evoked during your review. Best regards,

Dr Giulio Borghi

Reviewer #1:

All the grammatical corrections suggested have been made.

Reviewer #2:

INTRODUCTION

1. The number of homeless persons has been detailed as requested. The last available estimations were inserted as recommended by the editor in comment #1 in the introduction section. The new sentence is the following: “In the specific case of France, the last estimations made by the French Court of Auditors, had shown an increase in homelessness of nearly 110% between 2012 (143,000 people) and 2019 (300,000 people).” (lines 48-50).
2. The previously published research focuses on specific health outcomes (e.g., perinatal outcomes, food insecurity, mental health, etc.), on specific dimensions of homelessness (e.g., housing instability), or on a different population (e.g., overall homeless population). On the other hand, our research aims to provide a general description of the overall perceived impact of homelessness on health during pregnancy and the postpartum period, without focusing on a specific dimension of homelessness or on a specific health outcome, while prioritizing women’s perspective. We agree that data on the topic is not missing. We rather meant that a general description of this phenomenon from women’s perspective is not available yet and that it would be a helpful perspective in identifying possible solutions. For these reasons, we corrected the sentence (lines 65-67).

The study (<https://doi.org/10.1111/hsc.12972>) focusing on perceived barriers and facilitators in antenatal and postnatal healthcare access by homeless women has been integrated as suggested. On the other hand, the other reference (<https://doi.org/10.1016/j.ijnurstu.2021.103974>) was not cited as it focused on healthcare access of homeless women in general, and as we already included studies on this topic concerning our study population.

METHODS

1. The COREQ checklist was added in the supporting information (S1 Table). The checklist indicates in which page of the article each specific methodological aspect is provided.

PARTICIPANTS AND RECRUITMENT

1. Examples of who are people “from their social surroundings” have been added in lines 83-88. The choice of integrating people from women’s social environment was made to promote a global vision of the perceived impact of homelessness on the health of the study sample, and to reinforce the robustness of the results by cross-referencing the data collected during interviews. Nevertheless, this data was used as complementary material to further explore the studied phenomenon, being careful to respect women’s priorities and perspective. Moreover, as our research aimed at exploring how the social environment was perceived to impact the health of homeless women during pregnancy and the postpartum period, having the opportunity to interview people from their social surroundings helped us to better understand this phenomenon.
2. Details on how the use of ETHOS affected the recruitment of participants have been added in line 101-103.
3. Inclusion criteria have been detailed in lines 83-88.

DATA COLLECTION

1. The professional interpreters did not participate in the transcribing process. However, as the translation took place at directly during the interview, the interviewer made sure that the messages translated by the interpreters were correct by reformulating the answers in order to ask confirmation to participants directly during interviews. Moreover, results were confirmed by participants during the focus groups organised with those who accepted, during the first interview, to be informed about the results of the research.

DATA ANALYSIS

1. The coding tree has been added in the supporting information to provide further insights about data analysis (S1 Fig).
2. Further details about how the focus groups were integrated in the results have been added in line 159-161.

RESULTS

1. The dimensions of homelessness included in the ETHOS classification, as well as other factors related to them, were included as the final sub-themes, being the determinants of the health status perceived by women. We have prioritized a classification of results based on our study objective, which focused on women’s perception on how their health was impacted by homelessness. Moreover, some dimensions of homelessness, such as housing instability, could have an impact on more than one health dimension, creating difficulties on classifying the different perceptions of women. The inclusion of the coding tree in the supporting information provides a better view of the organization adopted and on the rationale behind this choice.
2. Quotes from participants have been added to the text as suggested.
3. Subsections of each dimension of health (mental, physical, social) have been created and the presentation of results has been rearranged accordingly.
4. Theme 2 and 3 have been combined as suggested.
5. Despite theme 4 contains less data than the others, we have chosen to keep it divided to emphasize the priorities of the study sample. The integration of the priorities in other sub-themes could reduce their relevance. Moreover, the topics highlighted as priorities could have a benefit on more than one dimension of health, making their classification under one theme difficult. We preferred to keep this theme because we used it in the discussion to explore possible solutions.

DISCUSSION

1. The comparison of our results with previously published research was done to further explore the themes evoked during interviews by adding insights found by previous research on that specific theme. This allowed us also to have a general picture on how the perception of the concerned population and of their priorities is aligned with previous research. The second part of the discussion was based on women’s priorities, exploring possible ways forwards, as suggested.
2. The adverse perinatal outcomes evoked as a consequence of homelessness have been detailed in lines 412-415.
3. The different forms of help have been detailed in lines 417-420.

CONCLUSIONS

1. The discussion has been restructured. In the conclusion section we provided a general resume of the findings and implications of this research.
